# Case Report: Catastrophic antiphospholipid syndrome in a pediatric patient after percutaneous treatment of aortic re-coarctation

**DOI:** 10.3389/fped.2024.1410400

**Published:** 2024-12-20

**Authors:** Iris Paola García Herrera, Carlos Canales Robredo, Magdalena Aboytes Zavala, Javier Merayo Chalico, Orfanel Sebastian Pineda Arzate, José Luis Colín Ortíz, Luis Alberto Aparicio Vera

**Affiliations:** ^1^ Pediatric Rheumatology Department, Hospital Para El Niño Poblano, Puebla, Mexico; ^2^ Pediatric Department, Hospital Para El Niño Poblano, Puebla, Mexico; ^3^ Rheumatology Department, Instituto Nacional de Ciencias Médicas y Nutrición Salvador Zubirán, Mexico city, Mexico; ^4^ Pediatric Cardiology Department, Hospital Para El Niño Poblano, Puebla, Mexico

**Keywords:** antiphospholipid antibody syndrome, catastrophic antiphospholipid syndrome, aortic recoarctation, heart disease, congenital heart disease

## Abstract

A female patient in middle childhood was diagnosed with coarctation of the aorta at one month of age and underwent a successful cortectomy. At 11 years old, she developed re-coarctation, which was managed through interventional cardiology. Shortly after the procedure, she experienced a sudden and severe clinical decline, presenting with hypoperfusion of the lower extremities, gastrointestinal bleeding, acute kidney injury, and pancreatitis. Multiple thrombotic events were identified, prompting an extensive evaluation for thrombophilia**.** The patient tested positive for antiphospholipid antibodies and was diagnosed with catastrophic antiphospholipid antibody syndrome (CAPS). An aggressive treatment was initiated, yielding a favorable response following discharge; she made a full recovery and continues to be monitored regularly in cardiology and rheumatology clinics.

## 1 Introduction

Antiphospholipid antibody syndrome (APS) is an autoimmune disorder characterized by the production of autoantibodies targeting membrane phospholipids or phospholipid-binding proteins, leading primarily to thrombotic events. Catastrophic APS (CAPS) is a rare but life-threatening variant of APS, marked by widespread thrombotic events with rapid progression, often resulting in multi-organ failure. Pediatric CAPS is associated with a high rate of mortality (33%–50%) (1). While some cases are triggered by infections or primary endothelial injury, this condition is rare in the pediatric population, particularly in patients with congenital heart disease. This makes this case unique in its presentation.

## 2 Case report

A 6-year-old girl with a history of aortic coarctation, diagnosed at one month of age, underwent surgical correction via termino-terminal coarctectomy. At 11 years old, she presented with re-coarctation, requiring replacement of Palmaz 4014 stent (Johnson and Johnson) at the site of the obstruction. The initial attempt via the right femoral artery was unsuccessful, leading to a second attempt through the left femoral artery using a 12 Fr Mullins introducer. Thirty-six hours post-procedure, the patient experienced sudden clinical deterioration, manifesting as vascular occlusion, with hypoperfusion of the lower extremities, gastrointestinal bleeding, acute kidney injury (Serum Creatinine 4.7 mg/dl), and acute pancreatitis (Serum amylase 629 U/L and serum lipase 1,745 U/L), D-dimer high levels (20,509 ng/ml) and liver transaminase levels (TGP 947 UI/L and TGO 4,819 UI/L) (Table 1).

**Table 1:** Approach to the diagnosis.

Laboratory study	Result/Units (reference value)	Result/Units (reference value)	Result/Units (reference value)
	First determination (at debut)	Second determination (12 weeks after)	1-year follow
Anti-cardiolipin antibodies			
IgG	2.77 mg/dl (<12 −, >12+)	3.77 mg/dl (<12 −, >12+)	8.77 mg/dl (<12 −, >12+)
IgM	<2 mg/dl (<12 −, >12+)	2.29 mg/dl (<12 −, >12+)	^a^ **14.20 mg/dl (<12 −, >12+)**
Anti-beta 2 glycoprotein 1 antibodies			
IgG	4.51 URml (<20 −, >20+)	4.51 URml (<20 −, >20+)	^a^**28.96 URml (<20 −,** >**20+)**
IgM	^a^**7.158 mg/L (**<**0.609 −,** >**2.366+)**	<0.5 mg/L (<0.609 −, >2.366+)	^a^**33.36 mg/L (**<**0.609 −,** >**2.366+)**
IgA	N/D	N/D	5.21 mg/dl (<10 −, >14.4+)
Lupus anticoagulant			
LA1 dRVVT	47.2 seg (31–44)	59.5 seg (31- 44)	47.1.5 seg (31–44)
LA2	34.1 seg (30–38)	35.9 seg (30–38)	34.2 seg (30- 38)
dRVVT	**1.4 (0.8–1.2)**	**1.7 (0.8–1.2)**	**1.4 (0.8–1.2)**
LA1/LA2^±^	^a^ **Presence of LA**	^a^ **Presence of LA**	^a^ **Presence of LA**
Antinuclear antibodies	Negative		
C3 complement	130 mg/dl (90–180)		
C4 complement	9.4 mg/dl (10–40)		

The bold values indicate positive and high values.

Lupic Antobody was determined using dilute Rusell Viper Venom test (DRVVT) technique, with LA1 screening, LA2 confirmatory and mixing test.

N/D, Not determinate.

^a^ Positive result.

One week after the catheterization, thromboses were identified in the bilateral common iliac arteries, left external iliac artery, external iliac vein, right femoral artery, bilateral renal arteries, pancreaticoduodenal artery, splenic artery, and superior mesenteric artery.

Unfortunately, no kidney biopsy or intestinal biopsy were obtained. Given the extensive thrombotic events, further investigation into thrombophilia was initiated. Inherited thrombophilia and non-autoimmune acquired thrombophilia (such as sepsis) were ruled out. However, the patient tested positive for antiphospholipid antibodies (Table 1), including anti-beta 2 glycoprotein and lupus anticoagulant, both with levels exceeding twice the laboratory-established cut-off values. Anti-beta-2 glycoprotein and anticardiolipin antibodies were measured using ELISA, and lupus anticoagulant was tested following the International Society of Thrombosis guidelines.

It should be noted that levels of both aPL antibodies fall below diagnostic thresholds despite the laboratory cut-off values. Despite this, we consider that there is an evident relationship between the positivity of the antiphospholipid antibodies and the clinical evolution of the patient.

A diagnosis of catastrophic antiphospholipid syndrome (CAPS) was made based on the Asherson Criteria. Treatment was initiated with high dose of methylprednisolone (1 g/day by 5 days), followed by prednisone, azathioprine, enoxaparin, and hemodialysis. After discharge from the intensive care unit (ICU) and internal medicine wards, a second positive lupus anticoagulant test was documented, and persistent antiphospholipid antibodies (for more than 12 weeks) confirmed the diagnosis of APS.

**Figure 1:**
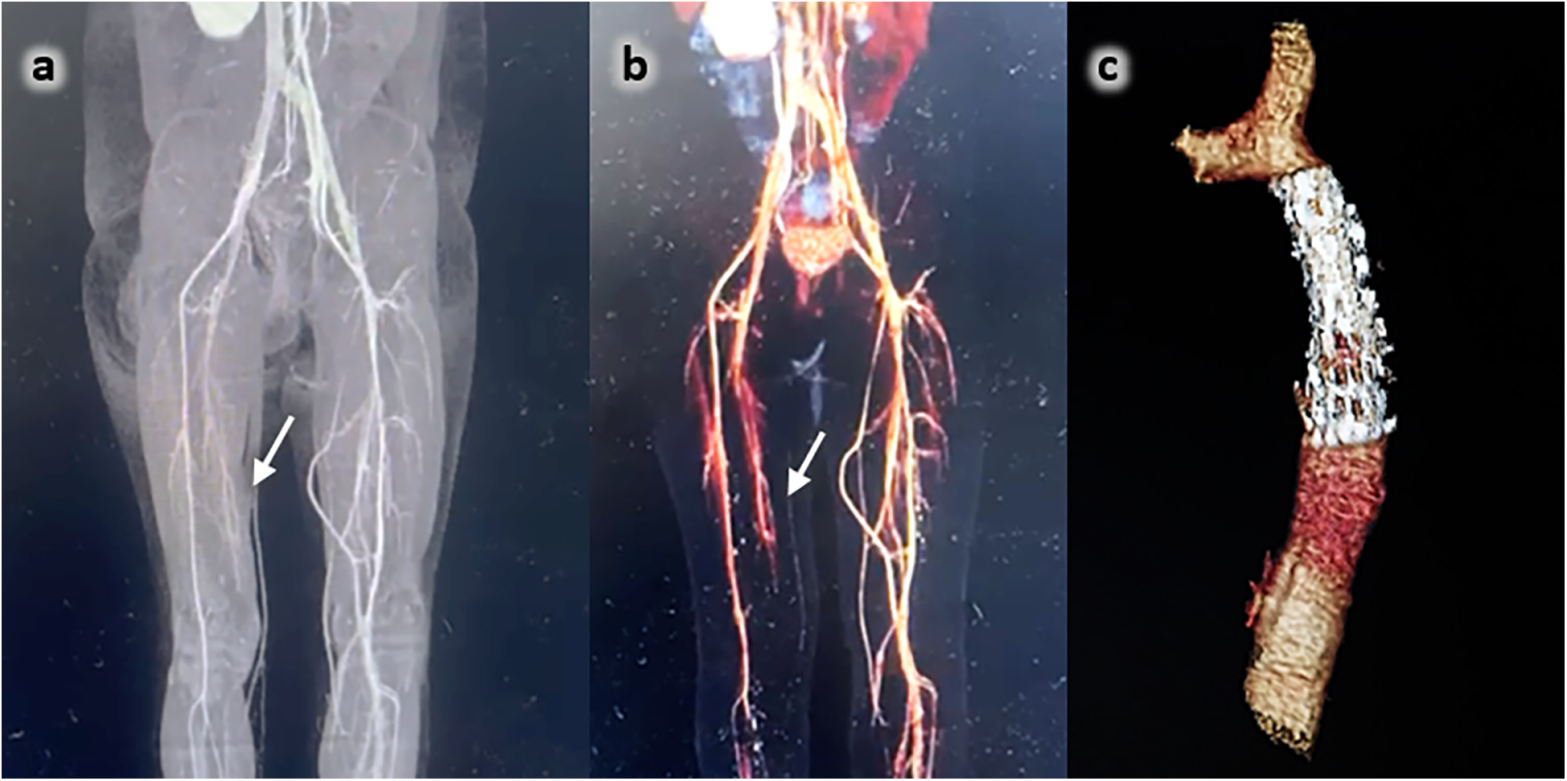
**(a,b)** Angiotomography of lower limbs: aneurysmatic dilatation and distal total occlusion of the internal right femoral artery (arrow). **(c)** Thorax 3D Angio tomography: The correct position of the stent inside the descending aorta.

One year post-diagnosis, the patient continued to test positive for antiphospholipid antibodies; two years after treatment, the patient's renal and pancreatic function have fully recovered; however, she has developed sequelae in her right lower limb due to total occlusion of the right femoral artery (Figure 1).

Today, the patient reports having a good quality of life, and a positive adaptation to her diagnosis. She and her family have adhered well to medical recommendations and treatment. Her current regimen includes hydroxychloroquine, gabapentin (for post-ischemic pain), aspirin, and acenocoumarol. Azathioprine was discontinued one year after the initial onset.

## 3 Discussion

APS is a systemic autoimmune disorder characterized by thrombosis associated with the presence of antiphospholipid antibodies (aPL). Pediatric antiphospholipid syndrome can occur at any age, from the neonatal period to adolescence (2). Catastrophic APS (CAPS) should be considered in patients with sudden thrombotic events affecting three or more organs or systems within a short period.

Pediatric CAPS has been reported in 10%–13% of patients with antiphospholipid syndrome (3). The CAPS diagnostic criteria were proposed by the International Task Force in 2003 and validated two years later (4). CAPS is frequently triggered by infections, trauma, or sepsis, with 60% of the cases in the pediatric population. No infectious agent was identified in our patient at the time of diagnosis. We hypothesize that the trigger was vascular trauma secondary to the cardiac catheterization procedure, which caused endothelial disruption. This scenario aligns with the “second-hit hypothesis” described in the pathogenesis of antiphospholipid syndrome.

We do not know if our patient had positive antiphospholipid antibodies before the cardiac catheterization. However, the literature indicates that approximately 11% of healthy children may have positive antiphospholipid antibodies. For thrombus formation to occur, a “second hit”, such as endothelial injury, infection, trauma, neoplasia, or an inherited prothrombotic risk factor, is usually required. The “second-hit model” suggests that the presence of aPL is necessary but insufficient to trigger the coagulation cascade without a secondary factor. In our case, the vascular trauma from the catheterization may have served as the “second hit”. In patients with immune dysregulation, this secondary event can precipitate thrombosis formation (5). Prior to surgery, the patient had no indications of autoimmune disease, thus, no aPL testing was performed. It is believed that thrombosis does not occur in patients without either vessel wall injury or the presence of antiphospholipid antibodies. Cardiac catheterization in pediatric patients is associated with a significant risk of arterial thrombosis (6). However, no studies have explored the relationship between APS and congenital heart disease to evaluate the risk of developing APS following invasive procedures such as catheterization. A review of the complications database at the Hospital for Sick Children in Toronto from 1994 to 2006 identified vascular complications as the most common adverse events during interventional procedures, with multiple thromboses reported. Risk factors included younger patient age (<6 months), male sex, inpatient status, and the age at catheterization. However, none of these patients tested positive for antiphospholipid antibodies (7).

To date, no case report has described CAPS or APS after catheterization procedures. This might be due to sudden death in some cases or the absence of serological screening before or after the interventions; additionally, diagnosis of CAPS requires a high index of suspicion. According to the International CAPS Registry, CAPS can be the first manifestation of APS in 86% of patients (8). CAPS can also occur in patients without a prior history of rheumatic disease. Freitas et al. reported CAPS in 76% of pediatric patients without a history of rheumatic disease following surgery (though not cardiac interventions) (9).

Our patient's case, where CAPS was the first manifestation of APS, aligns with findings from the CAPS Registry. APS diagnosis in pediatric patients requires updates, as current criteria may not fully apply to this population. Our patient met the Sidney Criteria for APS, with thrombosis plus persistently positive antiphospholipid antibodies; Even under the 2023 ACR/EULAR Antiphospholipid Syndrome Classification Criteria, our patient would be classified based on venous and arterial thrombosis plus persistent antiphospholipid antibodies (as assessed according to the methodology outlined in the criteria). From now on, there are no diagnostic criteria for CAPS in pediatric patients, and the diagnosis is still based on the 2003 criteria by Asherson et al. (10), later validated by Cervera et al. (4).

The most common systems in this life-threatening form of APS include the kidneys (73%), pulmonary system (60%), cardiac system (50%), and skin (47%) (11). In our patient, CAPS presented with peripheral thrombosis, kidney thrombosis, and gastrointestinal involvement (12). Gastrointestinal involvement, including pancreatitis and mesenteric thrombosis with intestinal bleeding, is uncommon in classic APS but more prevalent in CAPS, often associated with poor outcomes. Timely treatment is critical, involving anticoagulation, immunosuppression, and supportive therapy, the goal is to halt the inflammatory and immunological cascade, thus preventing fatal clinical manifestations.

The “triple therapy” approach, which includes glucocorticoids, anticoagulation with heparin, and therapeutic plasma exchange, is recommended, though not required for all patients; The SHARE initiative's recommendations for diagnosing and treating of pediatric CAPS advocate for triple therapy (13).

Our patient responded well to glucocorticoids, heparin, and immunosuppressants such as azathioprine, however, the need for hemodialysis was unavoidable. Rituximab and eculizumab have been considered for similar cases. Eculizumab has been proposed as a viable option for CAPS due to its ability to prevent the formation of the membrane attack complex (MAC) by inhibiting complement cleavage. Case reports suggest that eculizumab may improve outcomes in refractory CAPS cases, making it a potential alternative when rituximab or intravenous immunoglobulins are ineffective. However, eculizumab's high cost limits its accessibility. This medication was unavailable in our hospital, and the patient's family could not afford it. While it may have been a good option for refractory disease, our case did not require further treatment (14, 15).

## 4 Differential diagnosis

The development of thrombosis in children and adolescents is influenced by several risk factors, including (1) obesity, infections, sex, age, and genetic mutations in specific coagulation factors such as protein C, protein S, and Factor V Leiden (16, 17). Our patient tested negative for these thrombotic conditions, considering sex and age. Adolescent females are at an increased risk of developing thrombosis due to elevated estrogen levels, which enhance the production of thrombotic protein whole, decreasing (3) antithrombotic factors such as protein C, protein S, and antithrombin. Although our patient was female, she was not an adolescent; therefore, estrogen was not a contributing risk factor for her thrombosis, we also considered other pathological causes, such as protein C deficiency which is associated with thrombosis in large and small blood vessels. However, this condition did not entirely match our case, as patients with protein C deficiency typically develop intracranial lesions and/or purpura fulminans within the first two weeks of life. Our patient, being six years old and with no prior history of coagulation disorders, did not fit this profile. Additionally, protein C levels in our patient were within the normal range, leading us to rule out this condition (18).

## 5 Treatment and follow-up

The patient initially underwent surgical management for a termino-terminal coarctectomy; however, she later presented with aortic coarctation, necessitating reintervention via catheterization, during which angioplasty with a 6 × 12 × 40 mm stent was performed.

Upon diagnosis of CAPS, the patient was treated with a high methylprednisolone dose (30 mg.kg.day), hydroxychloroquine, azathioprine, enoxaparin, aspirin, and prednisone. The first-line treatment included enoxaparin along with a high-dose (bolus) intravenous methylprednisolone.

Subsequently, maintenance therapy was initiated with azathioprine, prednisone, acenocoumarol, and aspirin. The patient is undergoing treatment with anticoagulation therapy in conjunction with hydroxychloroquine (8).

## 6 Conclusion

This case is significant due to the rarity of catastrophic antiphospholipid syndrome (CAPS) associated with congenital heart disease in children. It supports the “double hit model”, highlighting the presence of antiphospholipid antibodies activated by thrombosis due to endothelial injury created during vascular access for interventional treatment. We believe this case report provides valuable insights for specialists in every clinical practice. Given the rarity of CAPS in the pediatric population, this condition may often misdiagnosed.

## Data Availability

The original contributions presented in the study are included in the article/Supplementary Material, further inquiries can be directed to the corresponding author.
